# Mitral isthmus block is associated with favorable outcomes after reablation for long‐standing persistent atrial fibrillation

**DOI:** 10.1002/clc.23415

**Published:** 2020-07-08

**Authors:** Xin‐hua Wang, Ling‐cong Kong, Zheng Li, Peng Nie, Jun Pu

**Affiliations:** ^1^ Department of Cardiology, Renji Hospital, School of Medicine Shanghai Jiao Tong University Shanghai China

**Keywords:** ablation < electrophysiology, arrhythmia/all, atrial fibrillation, clinical trials

## Abstract

**Background:**

Mitral isthmus (MI) ablation was limited due to technical challenges in the index ablation for long‐standing persistent atrial fibrillation (LPeAF). The role of adjunctive MI ablation was controversial.

**Hypothesis:**

MI block could be achieved in most patients undergoing repeat LPeAF ablation and was associated with favorable clinical outcomes.

**Methods:**

Of 87 consecutively patients undergoing reablation for recurrent atrial tachyarrhythmias (ATa), 41 patients with residual MI conduction but without pulmonary vein reconnection or left atrial roof conduction were enrolled to treat recurrent atrial flutter (AFL) (n = 20) and AF (n = 21). After AFL ablation and AF cardioversion, MI conduction gaps (CGs) were mapped and closed.

**Results:**

MI line was successfully blocked in 37 (90.2%) of 41 patients after closing 1.4 ± 0.5 CGs (31 endocardial CGs and 16 epicardial ones) in the initial MI lines. CGs were more often located at the endocardial sites close to the lateral ridge between left atrial appendage and left‐sided PVs, midportion of MI and at the epicardial breakthroughs within coronary sinus. At the end of 16.0 ± 1.9 months' follow‐up, 31 (83.8%) of 37 patients with MI block and 1 of 4 patients without MI block were free of further recurrence of ATa off anti‐arrhythmic drugs. MI block was positively associated with ATa‐free survival by Cox's regression analysis (hazard ratio [HR]: 0.012, 95% confidence interval [CI]: 0.000‐0.456, *P* = .02).

**Conclusions:**

MI block could be achieved in the majority of patients during repeat ablation for LPeAF. MI block was associated with favorable clinical outcomes after LPeAF reablation.

## 1 INTRODUCTION

Pulmonary vein isolation (PVI) is significantly less effective for long‐standing persistent atrial fibrillation (LPeAF) than for paroxysmal AF (PAF).^1^ The role of adjunctive linear ablation, complex fractionated atrial electrograms (CFAEs) ablation, and rotor ablation in LPeAF ablation is controversial.^2^, ^3^, ^4^, ^5^

The Cox Maze III procedure, by creating multiple incisions in both atria, is effective for treating lone AF or AF concomitant with valve replacement and other cardiac surgery.^6^, ^7^ It seems a “gap” existed between the efficacy of surgical incision lines and percutaneous ablation lines. One resonable explanation was the relatively low rate of line block in percutaneous ablation, since incomplete linear ablation is proarrhythmic.^8^, ^9^ The efficacy of catheter ablation for LPeAF might be improved if the lesion lines could be blocked.

It might be difficult to achieve a higher than usual rate of line block in the index procedures since aggressive or epicardial ablation might increase the risks of severe complications. However, in reablation it might be easier to achieve line block by closing gaps in initial ablation lines. In this study we hypothesized that mitral isthmus (MI) block could be achieved in the majority of redo LPeAF cases and MI block was associated with favorable outcomes.

## 2 METHODS

### 2.1 Patient population

From Jan 2018 to August 2018 patients undergoing reblation for recurrent atrial tachyarrhythmias (ATa) after initial catheter ablation of LPeAF were included retrospectively. LPeAF was defined as AF lasting for ≥1 year. The index ablation approach was circumferential PV isolation (CPVI) and linear ablation at left atrial (LA) roof and MI. Patients were eligible for enrollment in this study if they met the inclusion criteria: recurrent ATa after 3 months postablation; failure or intolerance to at least one antiarrhythmic drug (AAD); presence of residual MI conduction in reablation. The exclusion criteria were: LA thrombus detected by trans‐esophageal echocardiography (TEE); PV reconnection and/or residual LA roof conduction identified in reablation. This study was in accordance with the Declaration of Helsinki and was approved by the Institutional Ethnics Committee. All patients provided written informed consent.

### 2.2 Electrophysiological study

The reablation procedure was performed under conscious analgesia and sedation with continuous infusion of fentanyl and midazolam. All AADs were withdrawn for at least five half‐lives except amiodarone. Amiodarone was suspended for at least 1 month. A decapolar mapping catheter was positioned in coronary sinus via left subclavian vein access. Two Swartz sheathes(L1 type, St. Jude Medical, MN, California)were introduced from the right femoral vein and were advanced into the left atrium via two transseptal procedures. After PV venography, a duodecapolar mapping catheter (PentaRay, Biosense Webster, California) or a decapolar mapping catheter (Lasso Nav, Biosense Webster, California) was advanced into the LA for PV potential recording, LA geometry reconstruction and high‐density mapping. A 3.5 mm contact‐force sensing catheter (Smart‐touch Thermocool Navistar, Biosense Webster, California) was used for lesion creation under the guidance of CARTO 3 system (Biosense Webster).

### 2.3 AFL ablation and AF cardioversion

In patients with AFL, activation mapping and multiple pacing entrainment was performed to determine the reentrant circuit and the key isthmus. The entrainment site with the postpacing interval (PPI) < 20 ms longer than the AFL cycle length was deemed within the reentrant circuit.^9^ Linear ablation was applied at the isthmus to achieve AFL termination. After AFL termination, pacing maneuvers were applied to evaluate the integrity of the ablation lines.

In patients with AF, direct current cardioversion was performed to restore normal sinus rhythm (NSR).

### 2.4 Identification of residual MI conduction

**FIGURE 1:**
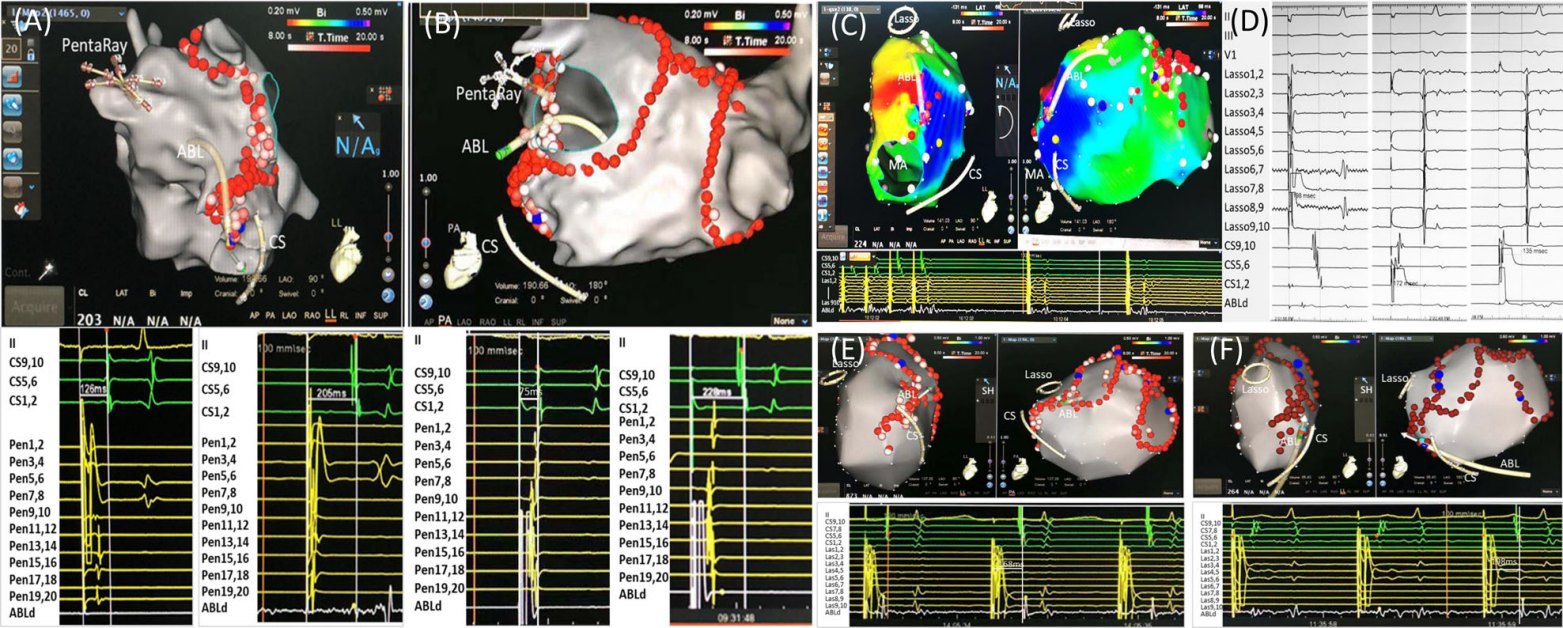
A,B: Evaluation of MI block by differential pacing from LAA and the ablation catheter (ABL) which was placed just lateral to the MI line. Initially LAA _pacing_ ‐ CSd interval was 126 ms with proximal to distal CS activation sequence (Low left panel of figure A), indicating possible MI block. However, ABL _pacing_ ‐ CSd interval was only 75 ms, which ruled out complete MI block (Low left panel of Figure B). After endocardial CG ablation close to the MA (blue dot), LAA pacing ‐ CSd interval was prolonged to 205 ms (Low right panel of Figure A), and ABL _pacing_ ‐ CSd interval to 228 ms (Low right panel of Figure B), indicating that MI was blocked. C: An example of peri‐mitral atrial flutter termination by endocardial CG ablation at the midportion (light blue dot) of MI line. D: During sinus rhythm, differential pacing showed that LAA _pacing_ ‐ CS1,2 interval was 198 ms with proximal to distal activation sequence of CS, CS1,2 _pacing_ ‐ LAA interval was 172 ms, and CS5,6 _pacing_ ‐ LAA interval was 135 ms, indicated achievement of bi‐directional MI block. E,F, Two examples of MI block achievement by endocardial CG ablation close to the ridge between LAA and LPV (site of ABL) and by epicardial CG ablation within the CS (light blue dot), noting the abrupt CS activation sequence change and the great prolongation of LAA _pacing_ ‐ CSd interval to 168 and 198 ms, respectively. ATa, atrial tachyarrhythmia; CG, conduction gap; CS, coronary sinus; LAA, left atrial appendage; LIPV, left inferior pulmonary vein; LSPV, left superior pulmonary vein; MA, mitral annulus

Residual MI conduction was deemed if: (a) the presence of peri‐mitral AFL; (b) multiple pacing maneuvors showed residual MI conduction during NSR. To check the presence of residual MI conduction, the ablation catheter was positioned just lateral to the MI line and the CS distal bipole (CSd) just septal to the MI line. The PentaRay or Lasso catheter was placed within the LAA. Residual MI conduction was deemed if at least one of the following criteria was not met: (a) By LAA pacing the CS conduction sequence was from proximal to distal (Figure 1A,E,F); (b) The interval from the ablation catheter pacing to CS distal bipole (CSd) was longer than that from LAA pacing to CSd (Figure 1A,B); (c) The interval from CSd pacing to the ablation catheter was longer than that from CS medium bipole pacing to the ablation catheter^10^ (Figure 1D).

### 2.5 Exclusion of PV reconnection and LA roof conduction recovery

PV reconnection was ruled out by absence of PV potentials or dissociation of PV potentials with the atrial electrograms^11^ in ongoing ATa, and was further excluded by both PV entrance and exit block in NSR. In NSR, LA roof conduction recovery was excluded if: (a) the activation sequence at the left atrial posterior wall (LAPW) was from caudal to cranial in NSR and during left atrial appendage (LAA) pacing; (b) the interval from LAA pacing to high LAPW was longer than that from LAA pacing to medium and low LAPW; (c) the interval from high LAPW pacing to LAA was longer than that from medium and low LAPW pacing to LAA.^12^, ^13^

### 2.6 Identification of conduction gaps in MI line

The endocardial conduction gaps (CGs) were deemed if: (a) fractionated potentials in previous MI lines and ablating such potentials resulted in peri‐mitral AFL termination; (b) the ablation sites with peri‐mitral AFL slowing or termination despite the absence of fractionated potentials (Figure 1C); (c) large bipolar potentials in the initial MI line during NSR or CS pacing; (d) the earliest activation site lateral or septal to the initial MI line during distal CS or LAA pacing^14^; (e) the sites where RF ablation resulted in prolongation of CSd‐LAA or LAA‐interval during CSd or LAA pacing. The epicardial CGs were considered at the earliest activation site within CS compared with the corresponding endocardial sites during LAA pacing^14^ (Figure 1F).

### 2.7 RF ablation settings

All ablation lesions were created by the contact‐force sensing saline‐irrigated catheter. For endocardial MI or LA roof ablation, radiofrequency (RF) energy was delivered at 35 to 40 W, 43°C and saline irrigation speed 20 mL/min, with the target ablation index (AI) 500 to 550. For epicardial ablation within the CS, RF energy was delivered at 25 W, 43°C and saline irrigation speed of 30 to 40 mL/min, with the duration of 30 seconds for each lesion.

### 2.8 Postablation management

All patients were kept on therapeutic oral anticoagulation for at least 3 months. NOACs (dabigatran or rivaroxaban) were preferred to warfarin unless they were contraindicated. Anticoagulation therapy were continued in patients experiencing further recurrence or in those free of recurrence but with high risk of thromboembolism (CHA2DS2VASc score >1 for male, or >2 for female), and could be withdrawn in patients free of recurrence and with low risk of thromboembolism. Amiodarone was continued for 3 months for all patients and was withdrawn if no further recurrence was detected, but could be continued otherwise. Oral proton‐pump inhibitors were administered for 2 months to reduce the probability of esophagus injury.

### 2.9 Follow‐up

The first 3 months postablation were defined as the blanking period of this study. Recurrences within the blanking period were treated by AADs or DC cardioversion. After the blanking period, the patients were followed up at the out‐patient clinic at 3, 6, 12, 18, and 22 months. During each clinic visit, ECG and 24‐hours Holter monitoring were performed to detect events of recurrence. The patients were asked to record their ECGs when there were symptoms indicating a recurrence. After the blanking period, the clinical success was defined as freedom from documented ATa recurrence (>30s) off AADs.

### 2.10 Data expression and statistical analysis

Continuous data were expressed as mean ± SD (SD) and category data as counts or proportions (%). Continuous data were compared by Student's *t*‐test if the variance was equal or by nonparameter test if the variance was unequal. Category data were compared by *χ* ^2^ or Fisher's exact test. The probability of ATa‐free survival in patients with AFL and AF recurrence was compared by Kaplan‐Meier analysis and Log‐rank test. Multivariable Cox proportional hazard regression model was applied to assess the factors associates with ATa‐free survival probability. A two‐tailed *P* < .05 was considered statistically significant. The statistical analysis was carried out by the SPSS 19.0 software (IBM, Armonk, New York).

## 3 RESULTS

### 3.1 Patients' baseline data

**FIGURE 2:**
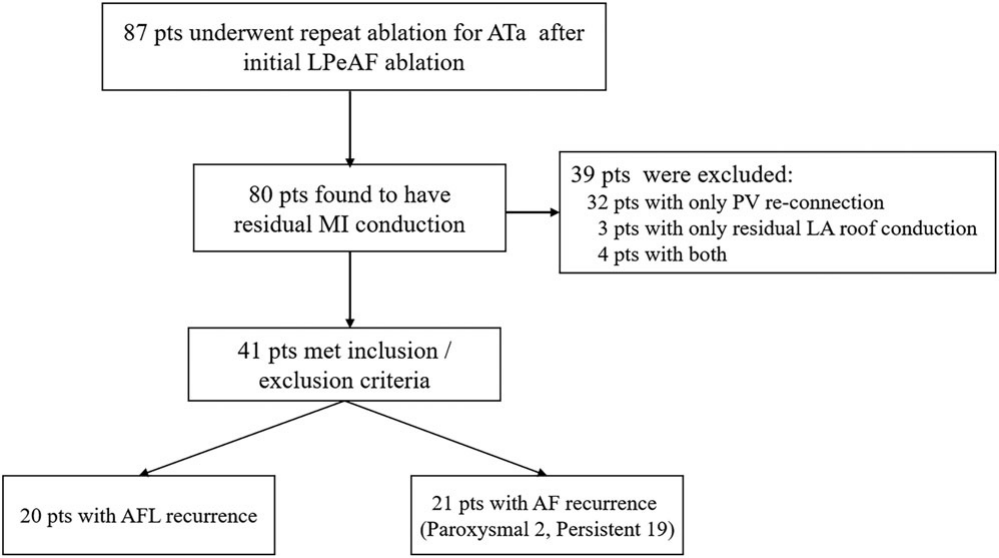
Patients' enrollment in this study

From Jan 2018 to August 2018 87 consecutive LPeAF patients (52 male, average age 67.4 ± 5.9 years) with ATa recurrence underwent reblation. During reablation, 41 patients with chronic PV isolation and LA roof block but without MI block met inclusion/exclusion criteria in this study (Figure 2). The types of recurrence were persistent AF in 19 patients, paroxysmal AF in two patients, and persistent atrial flutter (AFL) in 20 patients. The baseline clinical data were shown in Table 1.

**TABLE 1:** Patients' baseline demographic characteristics

Parameters	Value
Age (y)	65.4 ± 7.6
Male, n (%)	23 (56.1)
AF history (mo)	40.1 ± 29.8
Comorbidities	
Hypertension, n (%)	22 (53.7)
Hypertrophic Cardiomyopathy, n (%)	2 (4.9)
Diabetes mellitus, n (%)	6 (14.6)
Coronary artery disease, n (%)	3 (7.3)
Dual valve replacement, n (%)	2 (4.9)
Prior history of stroke	1 (2.3)
TTE measurement	
LAD (mm)	46.0 ± 4.4
LVEDD (mm)	49.4 ± 5.0
LVESD (mm)	34.4 ± 5.7
LVEF(%)	57.6 ± 8.6
Mitral regurgitation (mild to moderate)	10 (24.4)
Aortic regurgitation (mild to moderate)	5 (12.2)
Tricuspid regurgitation (mild to moderate)	8 (19.5)

Abbreviations: AF, atrial fibrillation; LAD, left atrial diameter; LVEDD, left ventricular end‐diastolic diameter; LVEF, left ventricular ejection fraction; LVESD, left ventricular end‐systolic diameter; TTE, transthoracic echocardiography.

### 3.2 Comparison between patients with recurrent AFL and AF

There was no significant difference in proportion of male, LAD, LV dimension, and LVEF between patients with AFL and AF recurrence. However, the average age was older and LAA blood flow velocity was faster in patients with AFL recurrence than in patients with AF recurrence (Table S1).

### 3.3 AFL mapping and ablation

AFL was present in 20 of 41 patients, including peri‐mitral flutter (PML) in 18 patients and typical cavo‐tricuspid isthmus (CTI) dependent AFL in 2 patients. In 18 patients with PML, AFL was terminated by closing endocardial gaps in 12 patients, and by epicardial ablation within CS in 4 patients. PML could not be terminated by endocardial and epicardial ablation in two patients. Typical AFL was terminated by CTI ablation in two patients. MI block was achieved in 17 of 20 patients, but was not achieved despite endocardial and epicardial ablation in the remaining three patients.

### 3.4 AF cardioversion and MI ablation

NSR was restored by DC shock in all 21 patients with recurrent AF. Endocardial CGs ablation achieved MI block in 15 patients, and additional epicardial ablation resulted in MI block in five patients. In the remaining one patient, MI failed to be blocked despite prolonged endocardial and epicardial ablation.

### 3.5 Characteristics of CGs ablation in initial MI line

**FIGURE 3:**
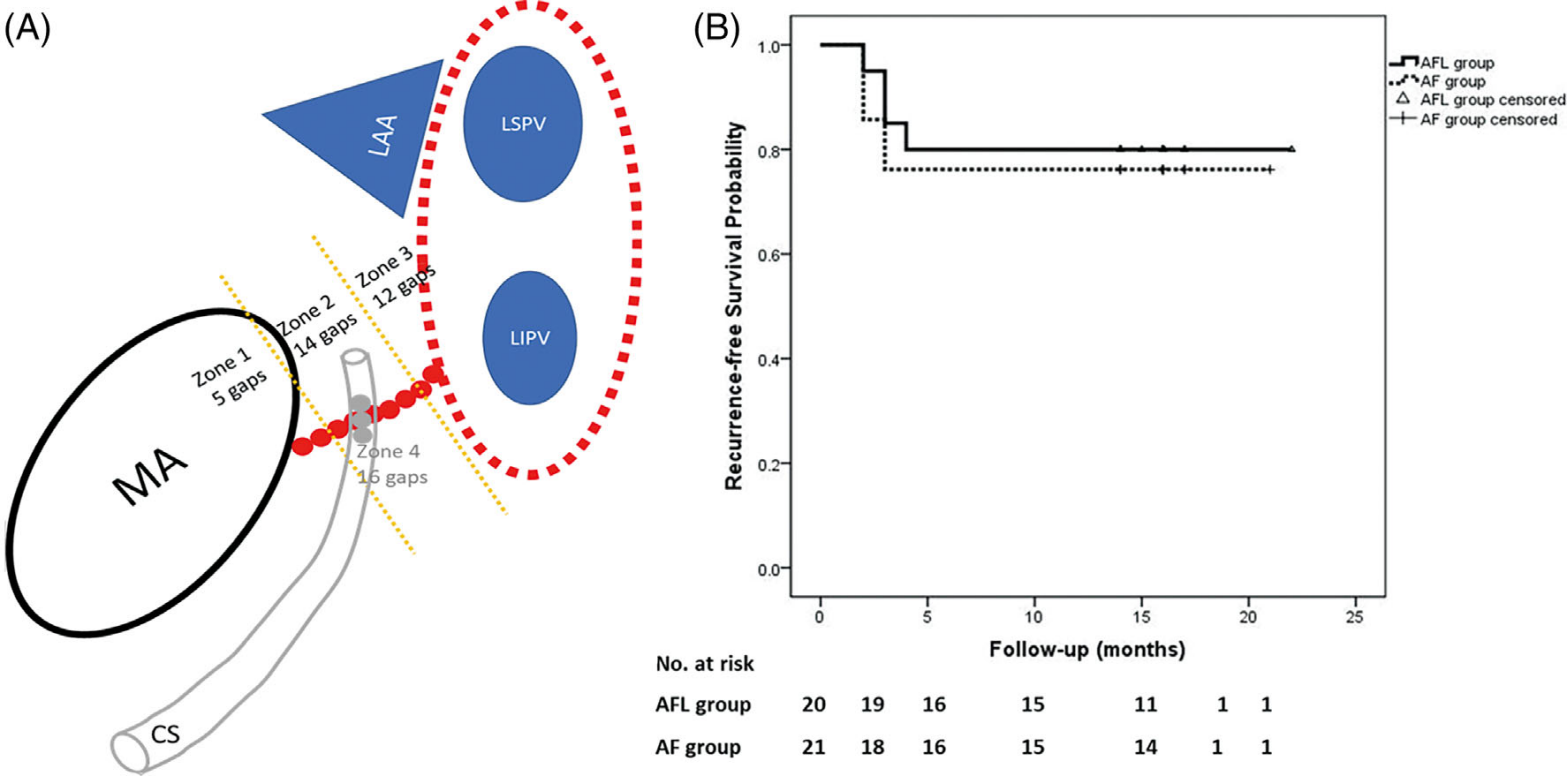
A, Anatomic distribution of CGs in the MI line during reablation, presented in left anterior oblique (LAO) view. Red dots represented endocardial MI ablation lesions. Red dotted circles indicated the circumferential PV lesions. Zones 1 to 3 represented the endocardial divisions of MI line. Each zone was separated by dotted yellow lines, with the number of CGs listed. Zone 4 represented the epicardial CGs with the coronary sinus (CS), which is shown in gray color. B, Comparison of ATa‐free survival probability following reablation between the patients with AFL and AF recurrence after the index procedure (Log rank test, *P* = .72). Abbreviations seen in Figure 1

The overall MI block was achieved in 37 (90.2%) out of 41 patients during reablation. There were 31 endocardial CGs and 16 epicardial ones, with an average number of CGs 1.4 ± 0.5 for each patient). The comparison of MI ablation in 41 patients at the index and reablation was presented in Table 2. The anatomic distribution of MI CGs was illustrated in Figure 3A. CGs were more often located at the endocardial sites close to the lateral ridge between LAA and LPV, midportion of MI line and at epicardial breakthroughs within CS.

**TABLE 2:** Comparison of MI ablation at the index and reablation procedures

Parameters	The index ablation	Reablation	*P* value
MI block, n (%)	3(7.3)	37 (90.2)	.00
Time for endocardial mapping and ablation (min)	23.8 ± 7.6	10.5 ± 4.6	.02
Proportion of ablation within CS, n (%)	27 (65.9)	28 (68.3)	.89
Time for mapping and ablation within CS (min)	8.5 ± 4.2	6.4 ± 3.5	.36
Total endocardial/epicardial lesions	615/156	362/124	—
Average number of lesions	18.8 ± 7.2	11.8 ± 9.5	.04
Total endocardial/epicardial CGs	—	31/16	—
Average number of CGs	—	1.4 ± 0.5	—
Total endocardial RF duration (min)	512	240	—
Total epicardial RF duration (min)	78	62	—
Average RT duration (min)	14.4 ± 5.2	7.4 ± 3.9	.01
Average AI for each endocardial lesions	524 ± 12	540 ± 18	.56
LAAp‐CSd interval (ms) after MI block	174.8 ± 10.9	182.1 ± 32.7	.23

Abbreviations: AI, ablation index; CG, conduction gap; CS, coronary sinus; LAAp, left atrial appendage pacing; MI, mitral isthmus; RF, radiofrequency.

### 3.6 Complications

No major complications (ie, tamponade, stroke, or atrio‐esophagus fistula) occurred. Coronary angiography following CS ablation showed no evidence of coronary artery injury.

### 3.7 Follow‐up data

At the end of 16.0 ± 1.9 (range 14‐22) months' follow‐up, 31 (83.8%) of 37 patients with MI block were free of further ATa recurrence off AADs. In contrast, 1 (25%) of 4 patients without MI block was free of further ATa recurrence. In 6 of 37 patients experiencing further ATa recurrence despite MI block (three with paroxysmal AFL and three with persistent AF). In three of four patients without MI block, there were two with persistent AFL and one with persistent AF.

After reablation, there was no significant difference in ATa‐free survival probability between patients with recurrent AFL and those with recurrent AF (80.0% vs 76.2%, Log rank test, *P* = .72, Figure 3B). Of the variables including age, AF duration, MI block and ultrasound parameters (LAD, LVEDD, LVEF, and LAA velocity), MI block was the only factor positively associated with ATa‐free survival (Hazard ratio: 0.012, 95% confidence interval: 0.000 to 0.456, *P* = .02, Table S2).

## 4 DISCUSSION

The major findings of our study were: (a) MI block could be achieved in the majority of redo cases despite block failure in initial ablation; (b) There was anatomic propensity of the conduction gaps in initial MI lines: the endocardial sites close to the lateral ridge between LAA and LPV, midportion of MI line and epicardial breakthroughs within CS; (c) MI block was associated with favorable clinical outcomes of LPeAF reablation.

### 4.1 Lessons learned from surgical Maze procedure

In the classic Maze III procedure, the “cut and sew” technique was utilized to create multiple incision lines, including circumferential PV incision, incision to the mitral annulus, LAA excision, incision from left‐sided PV to LAA, right atrial appendage excision, right atrial anterior incision, posterior incision to tricuspid annulus (TA), lateral incision from superior vena cava to inferior vena cava and adjunctive cryo‐lesion from mitral annulus (MA) to CS.^6^ The Maze incisions were devised to create atrial compartmentation preventing multiple wavelet reentries, and yielded a good long‐term success rate.^6^, ^7^ Bi‐atrial Maze was more effective than stand‐alone PVI,^15^ indicating the importance of AF substrate modification by linear incisions. Because the “cut and sew” technique was associated with elevated mortality, alternative energies (RF, cryo‐ablation, and microwave) were applied. However, non‐ “cut and sew” Maze was significantly less effective for durable block lines creation as well as NSR maintenance compared with “cut and sew” Maze,^16^ highlighting the importance of lines integrity for a good clincal outcome.

### 4.2 The challenges of MI ablation in the index procedure

It was technically challenging to perform MI linear ablation. Atrial tissue thickness, existence of pouch and MI length significantly affected the success rate of MI block.^8^, ^17^ To alleviate the “heat sink” effect resulting from the blood flow of the CS, CS balloon occlusion was reported to facilitate MI ablation. Epicardial ablation in the CS was needed in about 70% of patients to achieve MI block^10^; However, the epicardial breakthroughs might not be reached even by placing the ablation catheter deep within the CS. Ethanol injection into the VOM might be restricted by catheterization failure due to its anatomic variations.^18^

### 4.3 Improving MI line block by closing conduction gaps in reablation

Incomplete ablation usually resulted in tissue edema and swelling.^19^ The increased tissue thickness might prevent further energy penetration and resulted in failure of MI block. In the areas with incomplete ablation, tissue edema and swelling subsided after 2 to 3 months,^19^ and the tissue surviving the index ablation might form the CGs. In this study, the CGs often manifested as large or fractionated potentials during high‐density mapping, and could be precisely mapped with the earliest cross‐line activation by pacing maneuvers. In this scenario, it might be much easier to block the MI line by identifying and closing the CGs during reablation than the index ablation. We found that MI could be blocked in over 90% of patients with previous MI ablation failure in this study. Furthermore, we found the anatomic propensity of CGs in initial MI lines. The lateral ridge between LAA and LPV, midportion of MI line and epicardial breakthroughs within CS were the most common three locations for CGs, which provided useful information for rapid CGs localization and efficient MI ablation.

To be noted, according to the results of our study we pleaded for a two‐step approach for MI ablation if the MI line could not be easily blocked even after aggressive endocardial and epicardial ablation during the first ablation, since it could be more easily blocked by closing the conduction gaps at reablation than continuing to ablate the swollen tissue during the first ablation.

### 4.4 The impact of MI block on clinical outcomes of LPeAF ablation

The impact of MI block on clinical outcomes remained debatable. Willems and his colleagues^2^ found that linear ablation improved the success rate of persistent AF ablation. While linear ablation did not improve AF‐free survival in STAR AF II trial.^3^ To be noted, the success rate of linear ablation in the latter study was less than 80%. Due to the proarrhythmic property of incomplete ablation,^9^ the beneficial effect of linear ablation might be neutralized by its proarrhythmic effect. The poorer effectiveness of non‐ “cut and sew” Maze in contrast to “cut and sew” Maze also supported the importance of line integrity.^15^, ^16^ In our study, we found that MI block was exclusively and positively associated with ATa‐free survival after analysis of multiple variables, which indicating that MI block was associated with favorable clinical outcomes after reablation of LPeAF.

### 4.5 Limitations

This study was a retrospective, preliminary clinical study and enrolled limited cases of recurrence. Because MI was blocked in the majority of patients during reablation, the number of patients without MI block was not enough to compare the clinical outcomes between the patients with MI block and those without. Cox proportional hazard regression models could become unstable when the number of patients enrolled was low. The second limitation was the definition of clinical success was based on ECG, 24 hours Holter documentation during regular clinical visits and at the time when symptoms occurred, so asymptomatic or short episodes of ATa could not be ruled out. The third limitation was that although MI block was achieved in 37 of 41 patients, AFL or AF still recurred in 6 (16.2%) of 37 patients. The possible reasons were that AF substrate might not be sufficiently modified by multiple linear ablation, since less lines were drawn and LAA was intact in catheter ablation compared with surgical Maze procedure.

## 5 CONCLUSION

MI block could be achieved in the majority of patients during reablation. MI block was associated with favorable clinical outcomes after reablation for LPeAF.

## CONFLICT OF INTEREST

The authors report no relationships that could be construed as a conflict of interest.

## Supporting information


**Table S1** Comparison of demographic data between patients with AFL and AF recurrence

**Table S2** Cox regression analysis on the risk factors associated with ATa‐free survival
