# Supplementary material for: Mitral isthmus block is associated with favorable outcomes after reablation for long‐standing persistent atrial fibrillation
Source: Clin Cardiol. 2020 Jul 8;43(10):1119–25. doi: 10.1002/clc.23415 (PMC7533998; doi:10.1002/clc.23415)
Supplement: Supplementary file 1 — Table S1 Comparison of demographic data between patients with AFL and AF recurrence [file CLC-43-1119-s001.docx]

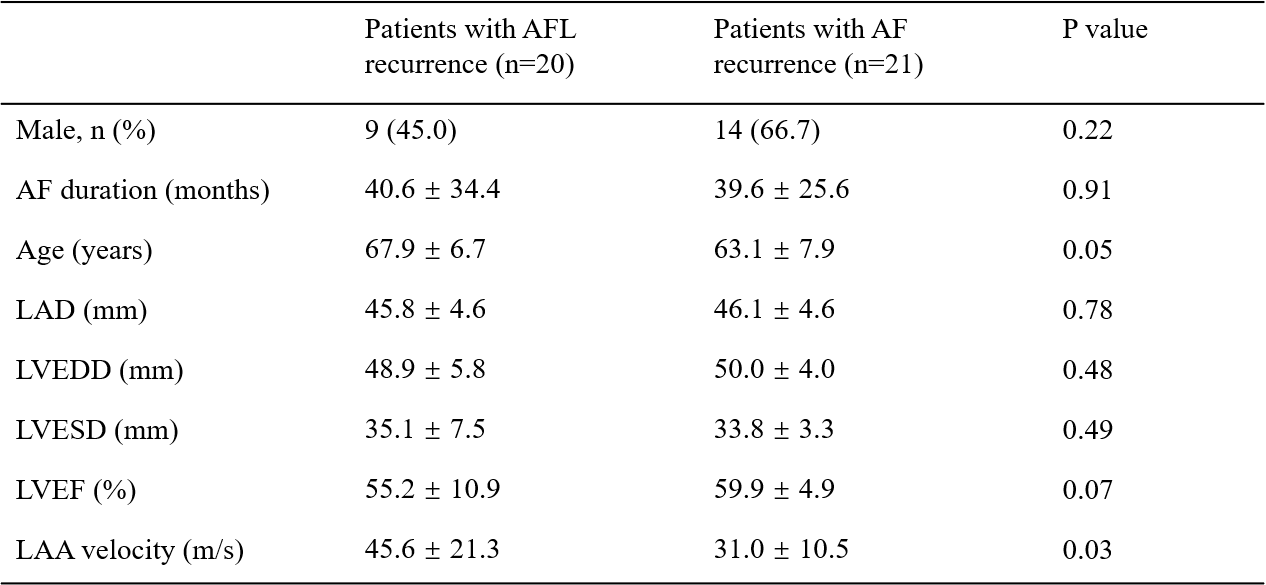


*AF=atrial fibrillation; AFL=atrial flutter; LAA=left atrial appendage; Ultrasound abbreviations seen in* ***Table 1.***

**Supplemental Table 1 Comparison of demographic data between patients with AFL and AF recurrence**
