# Supplementary material for: Mitral isthmus block is associated with favorable outcomes after reablation for long‐standing persistent atrial fibrillation
Source: Clin Cardiol. 2020 Jul 8;43(10):1119–25. doi: 10.1002/clc.23415 (PMC7533998; doi:10.1002/clc.23415)
Supplement: Supplementary file 2 — Table S2 Cox regression analysis on the risk factors associated with ATa‐free survival [file CLC-43-1119-s002.docx]

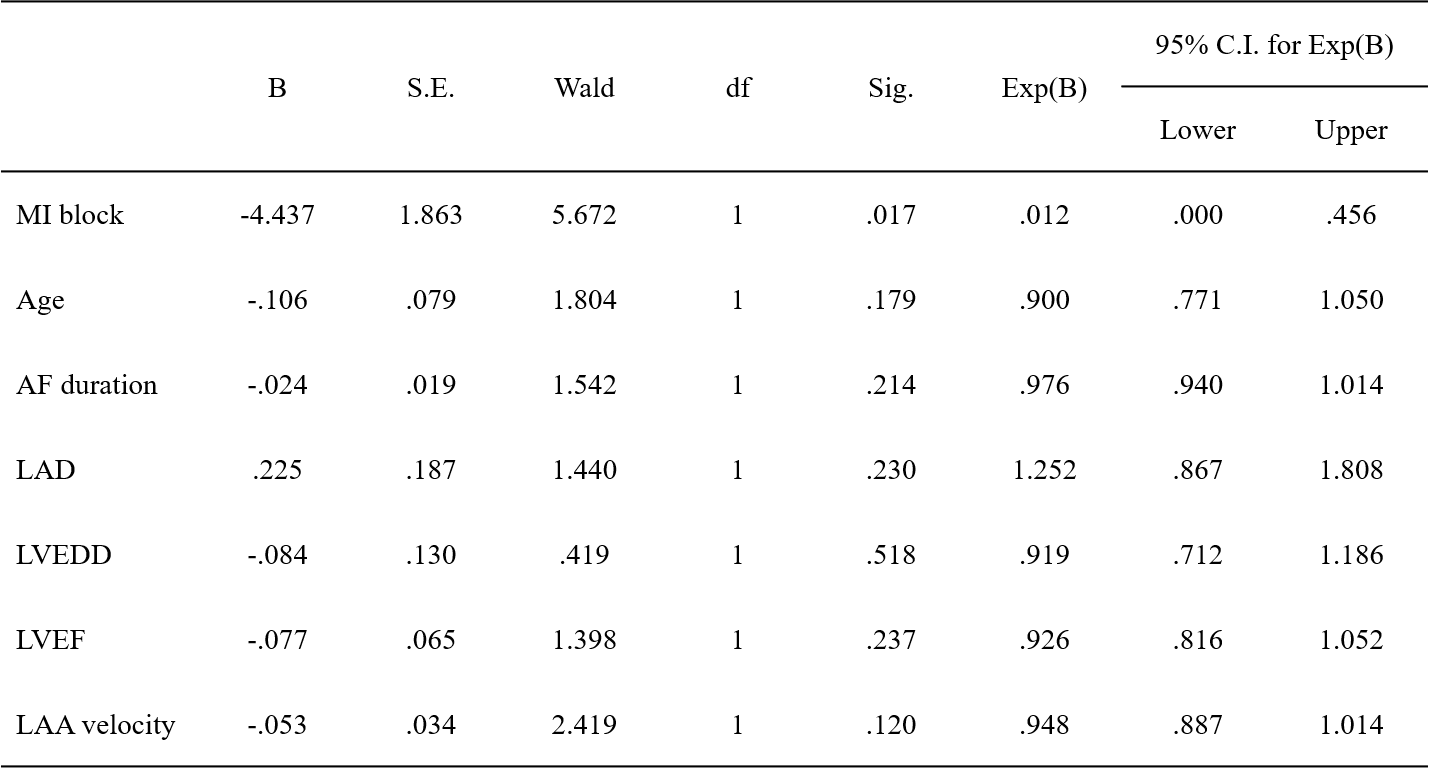


*MI=mitral isthmus; LAD=left atrial diameter;*

*LVEDD=left ventricular end-diastolic diameter; LVEF=left ventricular ejection fraction; LAA=left atrial appendage*

**Supplemental Table 2 Cox regression analysis on the risk factors associated with ATa-free survival**
